# Anticipated and current preventive behaviors in response to an anticipated human-to-human H5N1 epidemic in the Hong Kong Chinese general population

**DOI:** 10.1186/1471-2334-7-18

**Published:** 2007-03-15

**Authors:** Joseph TF Lau, Jean H Kim, Hi Yi Tsui, Sian Griffiths

**Affiliations:** 1 Centre for Epidemiology and Biostatistics, Faculty of Medicine, The Chinese University of Hong Kong, 5/F, School of Public Health, Prince of Wales Hospital, Shatin, N.T. Hong Kong, China; 2 School of Public Health, Faculty of Medicine, The Chinese University of Hong Kong, 5/F, School of Public Health, Prince of Wales Hospital, Shatin, N.T. Hong Kong, China

## Abstract

**Background:**

The prevalence of self-reported preventive behaviors in response to an anticipated local human-to-human H5N1 transmission outbreak and factors associated with such behaviors have not been examined.

**Methods:**

A random, anonymous, cross-sectional telephone survey of 503 Hong Kong Chinese adults.

**Results:**

The public in Hong Kong is likely to adopt self-protective behaviors (e.g., wearing face mask in public venues (73.8%), increasing the frequency of handwashing (86.7%)) and behaviors that protect others (e.g., wearing face masks when experiencing influenza-like illness (ILI, 92.4%), immediately seeking medical consultation (94.2%), making declarations when crossing the border with ILI (87.1%), complying to quarantine policies (88.3%)). Multivariate analyses indicated that factors related to age, full-time employment, perceived susceptibility, perceived efficacy of preventive measures, perceived higher fatality as compared to SARS, perceived chance of a major local outbreak, and being worried about self/family members contracting the virus were significantly associated with the inclination to adopt self-protective measures. Similar analyses showed that education level, variables related to perceived efficacy, perceived major local outbreak and such were significantly associated with various behaviors directed towards protecting others.

**Conclusion:**

In the event of a human-to-human H5N1 outbreak, the public in Hong Kong is likely to adopt preventive measures that may help contain the spread of the virus in the community.

## Background

As of December 7, 2006, bird-to-bird transmission of the H5N1 virus have been reported in over 50 countries [1], totaling 274 reported cases of bird-to-human transmission, with 167 resulting deaths [2]. From May to December, 1997, the first 18 cases of human H5N1 were reported in Hong Kong, resulting in 6 fatalities [2]. While human-to-human transmissions are considered rare occurrences [3,4], experts are concerned that a pandemic may occur via human-to-human transmissions [5,6], and it has been estimated that 7.4 million deaths may result worldwide [7].

During the SARS epidemic, preventive measures were commonly practiced [8] and a number of these behaviors [9-11] have been sustained by a large proportion of the public even after the SARS epidemic subsided in Hong Kong [12]. Preventive behaviors such as face mask use and handwashing have been suggested to be effective in the control of the SARS epidemic [9]. Understanding the correlates of these behavioral changes would facilitate formulation of policies and campaigns to promote appropriate behavioral responses in the event of a human-to-human H5N1 outbreak.

This study investigated the prevalence of self-reported preventive behaviors in response to a hypothetical local outbreak of human-to-human H5N1 transmission. These behaviors included both self-protective measures as well and those protecting others from contracting the virus. Factors associated with these behaviors were investigated.

## Methods

### Study population

The study population was comprised of male and female Hong Kong Chinese adults aged 18–60 years. An anonymous cross-sectional telephone survey using a structured questionnaire was conducted in November 2005 (n = 503). Table 1 summarizes the background characteristics of the respondents. Random telephone numbers were selected from up-to-date telephone directories. Telephone surveys have been used in a number of SARS and avian influenza studies [11-14]. Telephone calls were made by trained interviewers from 6:30 PM to 10:00 PM each night to avoid over-representation of unemployed persons. For unanswered calls, at least 3 other independent calls were made. The household member whose birthday was closest to the date of the interview was invited to join the study. Verbal informed consent was obtained from the respondents and ethics approval was obtained from the Ethics Committee of the Chinese University of Hong Kong. The response rate, defined as the number of completed interviews divided by the number of eligible households, was approximately 57%.

**Table 1:** Background characteristics of the respondents (n = 503)

	**Male**	**Female**	**All**
	(n = 234)	(n = 269)	(n = 503)
			
	Col%	Col%	Col%
**Age group**			
18 – 24	15.8	16.0	15.9
25 – 34	19.7	22.3	21.1
35 – 44	26.1	27.5	26.8
45 – 60	38.5	34.2	36.2
**Education level**			
Matriculated or below	61.8	68.8	65.5
College/university or above	38.2	31.2	34.5
**Marital status**			
Never married	40.2	34.9	37.4
Ever married	59.8	65.1	62.6
**Employment status**			
Not employed full-time	22.2	54.6	39.6
Employed full-time	77.8	45.4	60.4

### Measurements

Respondents were asked how likely they would be to adopt the following preventive behaviors if a local human-to-human H5N1 outbreak (defined as "if 2–3 new human-to-human transmission of H5N1 cases were to be reported in Hong Kong") were to occur: face mask use in public venues, increased frequency of handwashing, avoidance of eating poultry, declaration of influenza-like illness (ILI) symptoms at border health checkpoints, the seeking of medical consultation immediately with the onset of a fever, face mask use in public venues when having ILI symptoms and compliance with any quarantine policies. Responses were recoded into 2 categories (yes or no) from a 5-point Likert scale.

Respondents were asked about perceptions related to human-to-human H5N1 transmission, including perceived modes of transmission (whether human-to-human transmission of the H5N1 virus could occur via respiratory droplets, bodily contact, contaminated objects, eating well-cooked poultry), perceived susceptibility to H5N1 in different groups of people (self, family members, children, adults, older people, health care workers, food handlers, food vendors and the general public), perceived chance of having a major outbreak in Hong Kong in the next 12 months and perceived efficacy of various prevention measures (quarantine of infected people, face mask use in public venues, frequent handwashing, home disinfection, mass extermination of poultry). Respondents were also asked their perceptions of the current availability of effective treatments, whether they believed health outcomes would be worse than those of SARS (higher fatality and lower treatment efficacy) and the degree of concern they had about oneself or one's family contracting the virus if 2–3 new human-to-human H5N1 were to be reported in Hong Kong.

### Statistical methods

Univariate odds ratios of the associations between the studied perception variables and individual studied preventive behaviors were derived. Variables that were significant in the univariate analyses were further analyzed using multivariate logistic regression analyses. Statistical significance was set at p = 0.05 and SPSS software version 12.0.0 (SPSS Inc, Chicago, IL, 2003) was used for data analyses.

## Results

### Prevalence of protective behaviors in the event of a human H5N1 outbreak in Hong Kong

The majority would adopt self-protecting behaviors such as wearing face mask in public venues (73.8%), increasing frequency of handwashing (86.7%), avoiding eating poultry (63.8%), wearing face mask in public venues when having ILI symptoms (92.4%), seeing a doctor immediately when having a fever (94.2%), making declarations at border health checkpoints when traveling with ILI (87.1%), and full compliance with any quarantine policies (88.3%). Gender and educational differences were, in general, non-significant, whereas some significant age differences were noted. Overall, 41.6% of the respondents would practice all 7 types of protective behaviors (Table 2).

**Table 2:** Anticipated health-related behavioral responses if 2 to 3 new human-to-human H5N1 cases were to be reported in Hong Kong

	**Gender**			**Age group**			**Education level**			**All**
				
	**Male**	**Female**		**18 – 34**	**35 – 60**		**≤Matriculated**	**≥University**		
***(% Likely/very likely)^a^***	Col%	Col%	*p*	Col%	Col%	*p*	Col%	Col%	*p*	Col%
Face mask use in public venues	75.6	72.1	*.37*	65.1	78.9	***<.01***	75.4	70.5	*.24*	73.8
Increased frequency of handwashing	87.2	86.2	*.76*	81.7	89.6	***.01***	88.4	83.8	*.14*	86.7
Avoidance of eating poultry	61.1	66.2	*.24*	55.9	68.5	***<.01***	63.8	63.6	*.96*	63.8
Full compliance with any quarantine policies	86.8	89.6	*.32*	85.5	89.9	*.14*	87.5	89.6	*.50*	88.3
Face mask use in public venues if having ILI symptoms	90.6	94.1	*.14*	91.4	93.1	*.50*	90.6	96.0	***.03***	92.4
Declaration of ILI symptoms at border health checkpoints	89.3	85.1	*.16*	84.9	88.3	*.28*	86.3	88.4	*.50*	87.1
Seeking of medical consultation immediately with the onset of a fever	94.4	94.1	*.85*	95.2	93.7	*.50*	94.2	94.2	*1.00*	94.2
										
**Total number of the above anticipated behaviors**										
										
None	0.4	0.4	*.06*	1.1	0.0	***.01***	0.6	0.0	*.29*	0.4
1	0.4	0.4		0.5	0.3		0.6	0.0		0.4
2	1.7	1.9		3.2	0.9		2.1	1.2		1.8
3	6.4	3.0		4.8	4.4		4.0	5.8		4.6
4	2.6	7.8		6.5	4.7		4.0	8.1		5.4
5	16.7	18.6		20.4	16.1		17.9	17.3		17.7
6	34.2	26.0		33.9	27.4		31.6	26.0		29.8
All 7	37.6	42.0		29.6	46.1		39.2	41.6		40.0

^a ^Answer options include 'very likely', 'likely', 'unlikely', 'very unlikely' and 'not certain'.

### Perceptions related to anticipated human-to-human H5N1 outbreak in Hong Kong

The majority of the respondents believed that various groups of people would be highly susceptible to the virus. Of the respondents, 26.8% believed themselves and 33.6% believed their family members or the general public to be highly susceptible (Table 3). Over 90% of the respondents perceived "high"or "very high" efficacy in various preventive practices, and 71.8% perceived mass extermination of poultry as an efficacious measure. Of the respondents, 85.7%, 60.8%, 48.3% and 24.9%, respectively, believed that respiratory droplets, contaminated objects, body contacts and eating well-cooked poultry to be transmission modes of the virus (Table 3).

**Table 3:** Perceptions related human-to-human H5N1 infection

	**Gender**			**Age group**			**Education level**			**All**
				
	**Male**	**Female**		**18 – 34**	**35 – 60**		**≤Matriculated**	**≥University**		
	Col%	Col%	*OR*	Col%	Col%	*OR*	Col%	Col%	*OR*	Col%
**Perceived modes of transmission of human-to-human H5N1*(%Yes)***										
Respiratory droplets	85.0	86.2	*ns*	84.9	86.1	*ns*	87.2	82.7	*ns*	85.7
Bodily contact	50.4	46.5	*ns*	44.6	50.5	*ns*	47.1	50.3	*ns*	48.3
Objects contaminated with the virus	61.1	60.6	*ns*	63.4	59.3	*ns*	61.7	59.0	*ns*	60.8
Eating well-cooked poultry	22.2	27.1	*ns*	27.4	23.3	*ns*	24.0	26.6	*ns*	24.9
										
***No. of items with "yes" responses in the above 4 items***										
0 – 2	60.3	59.9	*ns*	60.2	59.9	*ns*	59.6	61.3	*ns*	60.0
3 – 4	39.7	40.1		39.8	40.1		40.4	38.7		40.0
										
**Perceived impacts of human-to-human H5N1**										
% Fatality rate worse than that of SARS	43.6	37.2	*ns*	36.0	42.6	*ns*	39.8	41.0	*ns*	40.2
% Efficacy of treatments worse than that of SARS	16.2	10.0	0.58*	12.4	13.2	*ns*	13.4	12.1	*ns*	12.9
% Perceived high/very high chance to have a major human-to-human H5N1 outbreak in Hong Kong	30.8	34.9		36.0	31.2	*ns*	33.7	31.2	*ns*	33.0
% Worry about oneself/family members contracting the virus^a^	47.4	59.1	1.60**	53.8	53.6	*ns*	54.1	53.2	*ns*	53.7
% Perceiving no effective drugs currently available	50.4	50.6	*ns*	54.8	47.9	*ns*	49.2	53.2	*ns*	50.5
										
**Perceived susceptibility to H5N1 infection*(% Likely/very likely)***										
Oneself or one's family members	27.4	26.4	*ns*	30.1	24.9	*ns*	22.2	35.8	1.96**	26.8
Older people	88.9	91.1	*ns*	93.5	88.0	0.51*	90.0	90.8	*ns*	90.1
Adults	49.6	46.8	*ns*	45.7	49.5	*ns*	44.7	54.9	1.51*	48.1
Children	84.6	86.2	*ns*	88.2	83.9	*ns*	85.7	85.5	*ns*	85.5
Health care workers	76.9	81.0	*ns*	83.9	76.3	0.62*	76.6	83.8	*ns*	79.1
Food sellers	82.9	87.4	*ns*	94.1	80.1	0.25***	80.9	94.2	3.86***	85.3
Food handlers	63.7	67.7	*ns*	74.2	60.9	0.54**	63.2	71.1	*ns*	65.8
The general public	35.9	31.6	*ns*	34.4	33.1	*ns*	30.4	39.9	1.52*	33.6
										
***No. of items with "likely/very likely" responses in the above 8 items***										
0 – 6	72.2	77.3	*ns*	71.5	77.0	*ns*	79.3	66.5	1.94**	75.0
7 – 8	27.8	22.7		28.5	23.0		20.7	33.5		25.0
										
**Perceived efficacy of prevention measures*(% High/very high)***										
Quarantine of the infected people	96.6	92.9	*ns*	94.1	95.0	*ns*	93.6	96.5	*ns*	94.6
Face mask use in public venues	90.2	90.7	*ns*	88.2	91.8	*ns*	91.2	89.6	*ns*	90.5
Frequent handwashing	93.2	94.1	*ns*	89.2	96.2	3.06**	94.2	93.1	*ns*	93.6
Home disinfections	90.2	90.3	*ns*	93.5	88.3	*ns*	90.6	90.2	*ns*	90.3
Mass extermination of poultry	73.9	69.9	*ns*	72.6	71.3	*ns*	71.4	72.8	*ns*	71.8
										
***No. of items with "high/very high" responses in the above 5 items***										
0 – 3	11.1	13.4	*ns*	14.0	11.4	*ns*	11.2	13.9	*ns*	12.3
4	28.6	32.0		29.6	30.9		31.0	29.5		30.4
5	60.3	54.6		56.5	57.7		57.8	56.6		57.3

^a ^Would worry that it is likely/very likely for oneself or one's family members to contract H5N1 (if 2–3 new cases were to be reported in Hong Kong.

OR = Univariate odds ratio. *p < 0.05; **p < 0.01; ***p < 0.001. ns = not significant.

With regard to the impact of the disease, 33% believed that there would be a major human-to-human H5N1 outbreak in Hong Kong in the coming year; 40.2% believed that it would have higher fatality as compared to SARS and 53.7% would be very worried about oneself or one's family members contracting the virus if an outbreak were to occur. Further, half of the respondents (50.5%) believed that effective drugs are now unavailable, fewer (12.9%) thought that the efficacy of treatment for this disease would be worse that that of SARS (Table 3).

### Factors predicting self-protecting behaviors if a local human-to-human H5N1 outbreak occurs

Certain factors were significantly associated with self-protective behaviors at the multivariate level. These include older age, full time employment, higher degree of perceived susceptibility, perceived efficacy of using face masks in preventing the disease, higher degree of perceived efficacy of the preventive measures, perceived higher fatality of H5N1 than SARS, anticipation of risk of a major outbreak in Hong Kong in the coming year, and concern about oneself/one's family in contracting the virus (OR = 1.86 to 2.78, p < 0.05, Table 4). Perceived efficacy related to mask use, handwashing and mass extermination of poultry, perceived higher fatality rate as compared to SARS and worry about oneself/one's family in contracting the virus were significant in the multivariate analysis in predicting perceived higher frequency of handwashing (OR = 1.94 to 7.76, p < 0.05, Table 4). Having ever been married, perceived susceptibility for food handlers, perceived efficacy of face mask use, perception that well-cooked poultry is a mode of transmission and perceived major outbreak in Hong Kong were multivariately predictive of avoidance of eating poultry (OR = 1.58 to 2.63, p < 0.05, Table 4).

**Table 4:** Factors associated with anticipated preventive behaviors protecting oneself if 2 to 3 new human-to-human H5N1 cases were to be reported in Hong Kong

	**Face mask use in public venues**			**Increased frequency of handwashing**			**Avoidance of eating poultry**		
			
	Row%	ORu	ORm	Row%	ORu	ORm	Row%	ORu	ORm
Background characteristics									
**Age groups**									
18 – 34	65.1	*1.00*	*1.00*	81.7	*1.00*	*ns*	55.9	*1.00*	*ns*
35 – 60	78.9	2.00**	2.38***	89.6	1.93*		68.5	1.71**	
**Marital status**									
Never married	64.9	*1.00*	*ns*	83.0	*1.00*	*ns*	55.3	*1.00*	*1.00*
Ever married	79.0	2.04**		88.9	***1.64^~^***		68.9	1.79**	2.11***
**Employment status**									
Not employed full-time	65.8	*1.00*	*1.00*	86.4	*ns*	*---*	61.3	*ns*	*---*
Employed full-time	78.9	1.95**	2.07**	86.8			65.5		
									
Perceived modes of transmission of human-to-human H5N1:									
**Respiratory droplets**									
No/not certain	62.5	*1.00*	*ns*	77.8	*1.00*	*ns*	61.1	*ns*	*---*
Yes	75.6	1.86*		88.2	2.13*		64.3		
**Bodily contact**									
No/not certain	70.0	*1.00*	*ns*	86.2	*ns*	*---*	61.2	*ns*	*---*
Yes	77.8	1.50*		87.2			66.7		
**Objects contaminated with the virus**									
No/not certain	72.1	*ns*	*---*	82.7	*1.00*	*ns*	62.4	*ns*	*---*
Yes	74.8			89.2	1.73*		64.7		
**Eating well-cooked poultry**									
No/not certain	73.8	*ns*	*---*	86.2	*ns*	*---*	60.3	*1.00*	*1.00*
Yes	73.6			88.0			74.4	1.91**	1.92**
**No. of items with "yes" responses in the above 4 items**									
0 – 2	68.9	*1.00*	*ns*	83.8	*1.00*	*ns*	60.6	*1.00*	*ns*
3 – 4	81.1	1.94**		91.0	1.97*		68.7	***1.42^~^***	
									
Perceived impacts of human-to-human H5N1									
									
**Fatality rate**									
Same as SARS/better than SARS/not certain	67.1	*1.00*	*1.00*	83.1	*1.00*	*1.00*	60.8	*1.00*	*ns*
Worse than SARS	83.7	2.51***	1.86*	92.1	2.37**	1.94*	68.3	***1.39^~^***	
**Efficacy of treatment**									
Same as SARS/better than SARS/not certain	71.5	*1.00*	*ns*	85.6	*1.00*	*ns*	62.8	*ns*	*---*
Worse than SARS	89.2	3.31**		93.8	***2.56^~^***		70.8		
**Perceived chance to have a major human-to-human H5N1 outbreak in Hong Kong**									
Low/very low chance/not certain	68.5	*1.00*	*1.00*	85.2	*ns*	*---*	59.9	*1.00*	*1.00*
High/very high chance	84.3	2.47***	2.23**	89.8			71.7	1.69*	1.58*
**Worry about oneself/family members contracting the virus^a^**									
Unlikely/very unlikely/not certain	63.9	*1.00*	*1.00*	81.5	*1.00*	*1.00*	57.5	*1.00*	*ns*
Likely/very likely	82.2	2.61***	2.11**	91.1	2.32**	2.20**	69.3	1.67**	
**No effective drugs currently available**									
Disagree/not certain	68.7	*1.00*	*ns*	83.5	*1.00*	*ns*	62.7	*ns*	*---*
Agree	78.7	1.69*		89.8	1.73*		65.0		
									
Perceived susceptibility to H5N1 infection									
									
**Oneself or one's family**									
Unlikely/very unlikely/not certain	69.3	*1.00*	*ns*	86.7	*ns*	*---*	62.5	*ns*	*---*
Likely/very likely	85.9	2.71***		86.7			67.4		
**Older people**									
Unlikely/very unlikely/not certain	64.0	*ns*	*---*	88.0	*ns*	*---*	56.0	*ns*	*---*
Likely/very likely	74.8			86.5			64.7		
**Adults**									
Unlikely/very unlikely/not certain	68.6	*1.00*	*ns*	84.3	*ns*	*---*	63.6	*ns*	*---*
Likely/very likely	79.3	1.76**		89.3			64.0		
**Children**									
Unlikely/very unlikely/not certain	61.6	*1.00*	*ns*	82.2	*ns*	*---*	67.1	*ns*	*---*
Likely/very likely	75.8	1.95*		87.4			63.3		
**Health care workers**									
Unlikely/very unlikely/not certain	63.8	*1.00*	*ns*	84.8	*ns*	*---*	64.8	*ns*	*---*
Likely/very likely	76.4	1.83*		87.2			63.6		
**Food sellers**									
Unlikely/very unlikely/not certain	68.9	*ns*	*---*	81.1	*ns*	*---*	45.9	*1.00*	*1.00*
Likely/very likely	74.6			87.6			66.9	2.38**	2.63***
**Food dealers**									
Unlikely/very unlikely/not certain	68.6	*1.00*	*ns*	86.0	*ns*	*---*	60.5	*ns*	*---*
Likely/very likely	76.4	***1.48^~^***		87.0			65.6		
**The general public**									
Unlikely/very unlikely/not certain	68.9	*1.00*	*ns*	86.2	*ns*	*---*	61.7	*ns*	*---*
Likely/very likely	83.4	2.28**		87.6			68.0		
**No. of items with "likely/very likely" responses in the above 8 items**									
0 – 6	69.0	*1.00*	*1.00*	86.2	*ns*	*---*	62.3	*ns*	*---*
7 – 8	88.1	3.33***	2.54**	88.1			68.3		
									
Perceived efficacy of prevention measures									
									
**Quarantine of the infected people**									
Low/very low/not certain	70.4	*ns*	*---*	88.9	*ns*	*---*	66.7	*ns*	*---*
High/very high	73.9			86.6			63.7		
**Face mask use in public venues**									
Low/very low/not certain	37.5	*1.00*	*1.00*	68.8	*1.00*	*1.00*	47.9	*1.00*	*1.00*
High/very high	77.6	5.77***	2.78*	88.6	3.52***	2.21*	65.5	2.06*	1.87*
**Frequent handwashing**									
Low/very low/not certain	68.8	*ns*	*---*	50.0	*1.00*	*1.00*	71.9	*ns*	*---*
High/very high	74.1			89.2	8.24***	7.76***	63.3		
**Home disinfection**									
Low/very low/not certain	63.3	*1.00*	*ns*	75.5	*1.00*	*ns*	63.3	*ns*	*---*
High/very high	74.9	***1.73^~^***		87.9	2.35*		63.9		
**Mass extermination of poultry**									
Low/very low/not certain	66.9	*1.00*	*ns*	78.9	*1.00*	*1.00*	56.3	*1.00*	*ns*
High/very high	76.5	1.61*		89.8	2.35**	2.12*	66.8	1.56*	
**No. of items with "high/very high chance" responses in the above 5 items**									
0 – 3	46.8	*1.00*	*1.00*	69.4	*1.00*	*ns*	*61.3*	*ns*	*---*
4 – 5	77.6	3.93***	2.75**	89.1	3.62***		*64.2*		

^a ^Would worry that it is likely/very likely for oneself or one's family members to contract H5N1 (if 2–3 new cases were to be reported in Hong Kong. Gender and education level were not associated with any of the 3 dependent variables and were hence not tabulated.

OR_U _= Univariate odds ratio.

OR_m _= Odds ratios obtained from multivariate stepwise logistic regression using univariately significant (including marginally significant) variables as candidate variables.

^~ ^0.5 < p < 0.1; *p < 0.5; **p < 0.01; ***p < 0.001.

ns = not significant.

--- univariately non-significant and was not considered in the multivariate stepwise logistic regression analysis.

### Factors predicting behaviors protecting others if an outbreak occurs

Multivariate results in Table 5 indicate education level, perceived efficacy related to face mask and perceived major outbreak in Hong Kong in the next year were predictive of anticipated use of face mask in public venues when having ILI symptoms (OR = 2.71 to 8.03, Table 5); perceived likelihood of a major outbreak in Hong Kong in the next year and perceived efficacy of disinfection of living quarters were multivariately associated with declaring ILI symptoms at cross-border checkpoints (OR = 3.09 and 2.87, respectively); perceived efficacy of mass extermination of poultry was the only factor predicting immediate doctor consultation when having fever (OR = 2.93). Ever-married status, perceived susceptibility related to children, perceived efficacy of face mask use were multivariately associated with intended full compliance with any quarantine policies (OR = 2.02 to 3.75).

**Table 5:** Factors associated with anticipated preventive behaviors to protect others if 2 to 3 new human-to-human H5N1 cases were to be reported in Hong Kong

	**Full compliance with any quarantine policies**			**Face mask use in public venues if having ILI symptoms**			**Declaration of ILI symptoms at border health checkpoints**			**Seeking of medical consultation immediately with the onset of a fever**		
				
	Row%	ORu	ORm	Row%	ORu	ORm	Row%	ORu	ORm	Row%	ORu	ORm
Background characteristics												
**Education level**												
≤ Matriculated	87.5	*ns*	*---*	90.6	*1.00*	*1.00*	86.3	*ns*	*---*	94.2	*ns*	*---*
≥ University	89.6			96.0	2.47*	2.99*	88.4			94.2		
**Marital status**												
Never married	83.5	*1.00*	*1.00*	91.0	*ns*	*---*	84.6	*ns*	*---*	94.1	*ns*	*---*
Ever married	91.1	2.02*	2.02*	93.3			88.6			94.3		
**Employment status**												
Not employed full-time	87.4	*ns*	*---*	88.9	*1.00*	*ns*	86.4	*ns*	*---*	92.0	*1.00*	*ns*
Employed full-time	88.8			94.7	2.24*		87.5			95.7	***1.96^~^***	
												
Perceived modes of transmission of human-to-human H5N1:												
**Respiratory droplets**												
No/not certain	79.2	*1.00*	*ns*	87.5	*1.00*	*ns*	90.3	*ns*	*---*	95.8	*ns*	*---*
Yes	89.8	2.32*		93.3	***1.98^~^***		86.5			94.0		
**Bodily contact**												
No/not certain	86.9	*ns*	*---*	91.9	*ns*	*---*	89.2	*ns*	*---*	93.8	*ns*	*---*
Yes	89.7			93.0			84.8			94.7		
**Objects contaminated with the virus**												
No/not certain	88.8	*ns*	*---*	91.4	*ns*	*---*	86.3	*ns*	*---*	93.4	*ns*	*---*
Yes	87.9			93.1			87.6			94.8		
**Eating well-cooked poultry**												
No/not certain	88.4	*ns*	*---*	91.8	*ns*	*---*	86.0	*ns*	*---*	93.9	*ns*	*---*
Yes	88.0			94.4			90.4			95.2		
**No. of items with "yes" responses in the above 4 items**												
0 – 2	87.7	*ns*	*---*	90.4	*1.00*	*ns*	87.1	*ns*	*---*	93.4	*ns*	*---*
3 – 4	89.1			95.5	2.27*		87.1			95.5		
												
Perceived impacts of human-to-human H5N1												
**Perceived chance to have a major human-to-human H5N1 outbreak in Hong Kong**												
Low/very low chance/not certain	86.6	*ns*	*---*	90.5	*1.00*	*1.00*	83.7	*1.00*	*1.00*	93.2	*ns*	*---*
High/very high chance	91.6			96.4	2.80*	2.71*	94.0	3.04**	3.09**	96.4		
**Worry about oneself/family members contracting the virus^a^**												
Unlikely/very unlikely/not certain	87.1	*ns*	*---*	89.7	*1.00*	*ns*	86.7	*ns*	*---*	92.7	*ns*	*---*
Likely/very likely	89.3			94.8	2.10*		87.4			95.6		
**No effective drugs currently available**												
Disagree/not certain	85.5	*1.00*	*ns*	91.6	*ns*	*---*	83.9	*1.00*	*ns*	92.8	*ns*	*---*
Agree	90.9	***1.70^~^***		93.3			90.2	1.75*		95.7		
												
Perceived susceptibility to H5N1 infection												
**Oneself or one's family**	87.2	*ns*	*---*	91.3	*ns*	*---*	85.3	*1.00*	*ns*	93.2	*ns*	*---*
Unlikely/very unlikely/not certain	91.1			95.6			91.9	***1.94^~^***		97.0		
Likely/very likely												
**Older people**												
Unlikely/very unlikely/not certain	78.0	*1.00*	*ns*	90.0	*ns*	*---*	86.0	*ns*	*---*	90.0	*ns*	*---*
Likely/very likely	89.4	2.38*		92.7			87.2			94.7		
**Adults**												
Unlikely/very unlikely/not certain	87.0	*ns*	*---*	91.6	*ns*	*---*	86.6	*ns*	*---*	94.6	*ns*	*---*
Likely/very likely	89.7			93.4			87.6			93.8		
**Children**												
Unlikely/very unlikely/not certain	78.1	*1.00*	*1.00*	90.4	*ns*	*---*	86.3	*ns*	*---*	91.8	*ns*	*---*
Likely/very likely	90.0	2.53**	2.50**	92.8			87.2			94.7		
**Health care workers**												
Unlikely/very unlikely/not certain	83.8	*ns*	*---*	91.4	*ns*	*---*	86.7	*ns*	*---*	93.3	*ns*	*---*
Likely/very likely	89.4			92.7			87.2			94.5		
**Food sellers**												
Unlikely/very unlikely/not certain	87.8	*ns*	*---*	85.1	*1.00*	*ns*	82.4	*ns*	*---*	89.2	*1.00*	*ns*
Likely/very likely	88.3			93.7	2.60*		87.9			95.1	2.36*	
**Food dealers**												
Unlikely/very unlikely/not certain	88.4	*ns*	*---*	88.4	*1.00*	*ns*	84.3	*ns*	*---*	94.2	*ns*	*---*
Likely/very likely	88.2			94.6	2.29*		88.5			94.3		
**The general public**												
Unlikely/very unlikely/not certain	86.2	*1.00*	*ns*	91.6	*ns*	*---*	85.6	*ns*	*---*	94.3	*ns*	*---*
Likely/very likely	92.3	1.92*		94.1			89.9			94.1		
**No. of items with "likely/very likely" responses in the above 8 items**												
0 – 6	86.7	*1.00*	*ns*	91.2	*1.00*	*ns*	85.7	*ns*	*---*	93.9	*ns*	*---*
7 – 8	92.9	***1.99^~^***		96.0	***2.32^~^***		91.3			95.2		
												
Efficacy of prevention measures												
**Quarantine of the infected people**												
Low/very low/not certain	74.1	*1.00*	*ns*	81.5	*1.00*	*ns*	81.5	*ns*	*---*	92.6	*ns*	*---*
High/very high	89.1	2.85*		93.1	3.05*		87.4			94.3		
**Face mask use in public venues**												
Low/very low/not certain	70.8	*1.00*	*1.00*	70.8	*1.00*	*1.00*	75.0	*1.00*	*ns*	87.5	*1.00*	*ns*
High/very high	90.1	3.75***	3.31**	94.7	7.40***	8.03***	88.4	2.53*		94.9	2.68*	
**Frequent handwashing**												
Low/very low/not certain	81.3	*ns*	*---*	84.4	*1.00*	*---*	78.1	*ns*	*---*	87.5	*ns*	*---*
High/very high	88.7			93.0	***2.46^~^***		87.7			94.7		
**Home disinfection**												
Low/very low/not certain	85.7	*ns*	*---*	87.8	*ns*	*---*	73.5	*1.00*	*1.00*	91.8	*ns*	*---*
High/very high	88.5			93.0			88.5	2.79**	2.87**	94.5		
**Mass extermination of poultry**												
Low/very low/not certain	84.5	*ns*	*---*	90.1	*ns*	*---*	88.7	*ns*	*---*	89.4	*1.00*	*1.00*
High/very high	89.8			93.4			86.4			96.1	2.93**	2.93**
**No. of items with "high/very high chance" responses in the above 5 items**												
0 – 3	77.4	*1.00*	*ns*	80.6	*1.00*	*ns*	77.4	*1.00*	*ns*	88.7	*1.00*	*ns*
4 – 5	89.8	2.57**		94.1	3.83***		88.4	2.23*		95.0	***2.42^~^***	

^a ^Would worry that it is likely/very likely for oneself or one's family members to contract H5N1 (if 2–3 new cases were to be reported in Hong Kong. Gender, age group, and perception that the impacts of human-to-human H5N1 would be worse than that of SARS in terms of fatality rate and efficacy of treatment were not associated with any of the 4 dependent variables and were hence not tabulated.

OR_U _= Univariate odds ratio.

OR_m _= Odds ratios obtained from multivariate stepwise logistic regression using univariately significant (including marginally significant) variables as candidate variables.

^~ ^0.5 < p < 0.1; *p < 0.5; **p < 0.01; ***p < 0.001.

ns = not significant.

--- univariately non-significant and was not considered in the multivariate stepwise logistic regression analysis.

## Discussion

These data indicate that the majority of the Hong Kong general public would adopt preventive measures, even in the event of 2–3 reported human-to-human H5N1 transmissions in Hong Kong. During the SARS epidemic, the prevalence of similar preventive behaviors increased sharply in the initial weeks of the outbreak [13].

Currently, we estimate that reasonably half of the general population is washing their hands over 10 times a day (unpublished data). An even higher frequency of handwashing is expected if the anticipated outbreak occurs. This is consistent with the local government campaigns for promoting handwashing [15]. Handwashing has been efficacious in preventing influenza [16,17] and SARS [9] and the vast majority of the respondents (94%) believed that it would be efficacious in preventing human-to-human avian influenza. Such a belief was, in turn, associated with anticipated higher frequency of handwashing. Handwashing may have become a commonly accepted means of preventing infectious respiratory diseases. Approximately 90% of the general population reported face mask use in public venues during the peak of the SARS epidemic [12,13]. The majority of the respondents would do so if there was a human-to-human H5N1 outbreak in Hong Kong. Many believed that face mask use in public venues was an efficacious method of human-to-human H5N1 prevention. However, while 87.1% of the respondents reported that they would report ILI symptoms at health checkpoints as required at times of a human-to-human H5N1 outbreak, 70.8% of the respondents did not do so during the period of April 2003 to January 2004 (during and shortly after the SARS epidemic) (unpublished data) [8]. Studies have demonstrated the limited effectiveness of such measures implemented during the SARS epidemic [18,19]. It is shown that about 90% of the general population with ILI symptoms during the SARS epidemic were wearing face masks [13], which is comparable to the results of this study showing that about 92% would wear face masks. The similarities between various public health responses related to SARS and avian influenza are reported and it is speculated that the public is modeling their responses to avian influenza outbreak to those of the SARS epidemic.

During the SARS epidemic, the reported prevalence of face mask use when having ILI while traveling abroad was not very high, indicating that a substantial proportion of the general population was not practicing behaviors directed towards protecting others [10]. This is in contrast to the results of this study, which noted very high proportion of respondents stating their intent to adopt of behaviors directed towards protecting others. The perceived severity of H5N1 may elicit more behaviors directed at protecting others.

Quarantine was an effective means contributing to the control of SARS [20,21] but a comparatively low percentage of the respondents (73.9%) believed quarantine to be an effective public health measure for control of avian influenza, and 11.7% of the respondents would not fully comply with government quarantine policies indicates there is still a need for education. As perceived efficacy was univariately associated with anticipated compliance with any quarantine policies, dissemination of information about its efficacy may be useful. Single respondents were less likely to comply fully with quarantine policies are also generally more mobile. These people may have less support if quarantined, and this should be considered by policy makers.

From 2003 until the present, the reported fatality rate of avian influenza in human has been over 50% [22], which is markedly higher than the 9.6% case fatality of rate SARS [23]. However, only about 40% of the study's respondents considered human-to-human H5N1 fatality rate to be higher than that of SARS. Hence, the actual magnitude of behavioral responses might be even stronger, since we found perceived fatality to be significantly associated with self-protective behaviors. It is particularly interesting to note that while associations with self-protective behaviors such as mask use and handwashing were highly significant, perceived fatality rate in comparison to SARS was not significantly associated with any of the 4 behaviors directed to protecting others. Therefore, different considerations may be involved in making decisions of whether to practice preventive behaviors, depending on whether such behaviors are self-directed or directed towards others.

With full population compliance with quarantine policies, the critical battle front of the avian influenza epidemic would shift to effective hospital infection control. It is unlikely that health care workers will be able to comply to all the stringent occupational guidelines in the event of nosocomial human H5N1 outbreaks. High fatality rates may occur in these health settings if panic or widespread non-adherence to safety measures occur. Research, counseling and emergency plans are essential to ensure that front-line health care workers are psychologically prepared and that the operations of health systems will not be disrupted.

The public regarded older people, children, health care workers and food vendors as particularly at risk of contracting the virus. During the SARS epidemic, it was reported that discriminatory attitudes have been expressed toward some at-risk groups, such as health workers [24,25]. It is worth noting that certain social groups, such as health care workers or food vendors, may be stigmatized similarly during an H5N1 influenza. Preventing or minimizing this should thus be focus of future study.

Approximately one-quarter of the respondents believed that they or their family members would likely/very likely be affected by an H5N1 outbreak whereas approximately one-third of the respondents believed this to be true for the general population. It was reported that during the SARS epidemic, the general public worried about themselves or their family members' contracting the virus [13]. This is expected to be repeated if a human-to-human avian flu outbreak occurs. These perceptions were also associated with anticipated preventive behaviors. Many studies have documented severe distress in the community during and after the SARS epidemic [11,13] and attention should be given in reduce panic at times of a human-to-human H5N1 outbreak. With the potentially high fatality and infectivity, high level of distress in the public is expected.

Although prevalence of health-seeking behaviors are usually higher among females than males, gender was not associated with any of the studied behaviors in this study. The health threat in this case may have overridden the aforementioned gender differentials. Education level was not associated with self-directed preventive behaviors but was associated with one of the others-directed preventive behaviors. It is possible that altruism is associated with education level. The reverse was true with age. Another study indicated that higher age was associated with the more use of preventive measures [26].

In general, factors related to perceived susceptibility, perceived clinical severity of outcomes (e.g. fatality rate, perceived availability and efficacy of treatments) were associated with anticipated preventive behaviors. Such variables are the key factors prescribed by the Health Belief Model (HBM), which stated that adoption of health behaviors is a function of an individual's attitudes and beliefs about the health issue/behavior of concern [27]. These variables were significant in predicting preventive behaviors related to SARS and influenza [9,12,13]. The HBM is therefore applicable to understanding behaviors for preventing the spread of emerging infectious diseases.

The study has a number of limitations. First, the study was conducted using telephone surveys and some households may not have been included. In Hong Kong, however, almost all households have telephones [28] and a large number of local published studies on SARS [11] and avian influenza [11,13,14] have utilized this method. Second, the response rate of the study was not very high. Nevertheless, the response rate was similar to many of other published local studies [14,28,29]. with the distributions of 18–39 and 40–60 year age groups being very comparable to those obtained from the Census data (49.6% and 50.4%, respectively). The study's gender distribution was also comparable to the Census distributions (47.8% male and 52.0% female) [30]. Adoption of behavioral responses was self-reported and had not been validated. However, during this pre-outbreak stage, it is unlikely that social desirability strongly biased the reporting of these behaviors.

While the results of this study may have meaningful regional policy implications, caution should be given when generalizing the results of this study to other countries. It is less clear whether populations which were relatively unaffected by SARS and populations that demonstrated less frequent face mask use and other relevant public health measures would exhibit the same magnitude of intended behavioral responses as the population of Hong Kong. Cultural and perceptions factors (such as perceived efficacy of prevention means) would also result in different prevalence of behavioral responses. International comparisons are therefore also greatly warranted.

## Conclusion

In the event of a human-to-human H5N1 outbreak, the Hong Kong public is very likely to adopt strong preventive measures in order to protect themselves and others. The magnitude of these behavioral responses may be even greater than those witnessed during the SARS epidemic and would be likely to increase if a high fatality rate or high infectivity rate were reported. These preventive behaviors may be an effective firewall to the continued spread of the virus in the community. Surveillance of public responses is an integral part of the government's H5N1 preparedness plan. It should address issues related to the potentially underestimated fatality associated with human H5N1 cases. Surveillance should also closely monitor prevalence of important public health behaviors such as quarantine compliance and cross-border preventive measures. Up-to-date surveillance information is necessary for the government to implement and make rapid adjustments these public health measures.

## Competing interests

The author(s) declare that they have no competing interests.

## Authors' contributions

JL conceptualized, oversaw the project, drafted and made final revisions of the manuscript. JHK helped to draft the manuscript and assisted in the statistical analysis. HYT participated in the design and coordination of the study and performed the statistical analysis. SG was involved in the conceptualization of the study andediting of the manuscript. All authors read and approved the final manuscript.

## Pre-publication history

The pre-publication history for this paper can be accessed here:


